# The Impact of the COVID-19 Pandemic and Lockdown on Adult Foot and Ankle Fractures Presenting to the Largest Trust in the United Kingdom

**DOI:** 10.7759/cureus.48262

**Published:** 2023-11-04

**Authors:** Upamanyu Nath, Amir Reza Akbari, Benyamin Alam, Rohan Dahiya, Anand Pillai

**Affiliations:** 1 Trauma and Orthopaedics, Wythenshawe Hospital, Manchester, GBR; 2 Medicine, King's Mill Hospital, Sutton-in-Ashfield, GBR; 3 Otolaryngology, Queen Elizabeth Hospital Birmingham, Birmingham, GBR; 4 Internal Medicine, Wythenshawe Hospital, Manchester, GBR

**Keywords:** post-lockdown, covid-19 lockdown, ortho surgery, foot, ankle, nhs, ankle fractures, foot & ankle surgery, foot and ankle fracture, covid 19

## Abstract

Background

The COVID-19 pandemic induced unprecedented changes in medical practices, prompting a reassessment of their impact on adult foot and ankle fractures within the National Health Service (NHS). This study employs a retrospective observational approach, leveraging the Pathpoint™ eTrauma platform for a comprehensive analysis of prospectively collected data.

Methods

Data encompassing weekly fracture incidence, weekly surgical procedures, patient demographics, and mean wait time from injury presentation to surgery were systematically evaluated. The study population included all adults (18+) admitted during five distinct periods: pre-pandemic, national lockdown 1, post-lockdown, national lockdown 2, and national lockdown 3.

Results

An analysis of 434 foot and ankle fractures revealed that national lockdown 1 exhibited the lowest fracture incidence (4.97 per week) and surgeries performed (4.77 per week), reflecting a notable reduction in trauma cases and elective procedures. Conversely, post-lockdown displayed the highest fracture incidence (7.46 per week) and surgeries performed (6.31 per week), suggesting a resurgence in both trauma and elective surgical activities. The pre-pandemic cohort, characterized by the highest mean age (51.98 years) and mean wait time (8.74 days), served as a temporal baseline.

Conclusion

While the incidence of fractures decreased during all three national lockdowns compared to pre-pandemic or post-lockdown periods, a gradual increase was observed in subsequent lockdowns. Notably, mean wait times showed a significant reduction, reaching the lowest point (5.79 days) during national lockdown 3. These findings underscore the complex interplay between pandemic-related disruptions, evolving guidelines, and adaptive measures within the healthcare system, influencing the dynamics of foot and ankle fracture management.

## Introduction

Background 

The coronavirus disease 2019 (COVID-19), arising from the severe acute respiratory syndrome coronavirus 2 (SARS-CoV-2), emerged in December 2019 following instances of pneumonia of unknown origin in Wuhan, China [1]. Rapidly escalating, the World Health Organization (WHO) officially categorized COVID-19 as a global pandemic on March 11, 2020 [1]. The United Kingdom (UK) responded promptly, implementing a nationwide 'lockdown' on March 24, 2020, aimed at mitigating transmission and safeguarding the National Health Service (NHS) [2]. Stringent measures, such as the closure of non-essential establishments, limitations on social gatherings, and the suspension of major sporting events, were enforced, with citizens advised to work from home and venture outside solely for essential needs [2].

Concomitant with the imposition of lockdown measures, the NHS underwent significant transformations [3]. Elective surgical procedures across all specialties were deferred to augment bed capacity and conserve personal protective equipment [3]. Additionally, staff redeployment, routine appointment cancellations, and diminished emergency department presentations were observed [4]. Despite these interventions, the WHO reported that as of April 30, 2021, 4,416,623 confirmed cases and 127,517 deaths were attributable to COVID-19 in the UK [5].

Profound concerns persist regarding the enduring repercussions of pandemic-induced disruptions in healthcare delivery, particularly pertaining to the NHS and individual well-being [4]. Staff shortages, procedural backlogs, and healthcare disparities have intensified, with the protracted consequences of these disturbances yet to fully manifest. This article delineates a focused exploration of the impact of the COVID-19 pandemic, associated lockdown, and subsequent modifications in NHS protocols on patients afflicted with foot and ankle fractures.

Foot and ankle fracture guidelines 

Patients presenting with foot and ankle fractures necessitate a nuanced approach, either conservative or surgical, contingent upon the fracture type. According to the British Orthopaedic Association standard for trauma (BOAST), stable fractures warrant analgesia and splinting, with patients permitted to bear weight as tolerated [6]. Conversely, BOAST and the National Institute for Health and Care Excellence (NICE) recommend surgical fixation for unstable fractures [6]. Optimal surgical intervention, ideally within 24 hours of injury, correlates with enhanced patient outcomes [6, 7]. Nevertheless, practical constraints often result in surgery within two to 14 days post-injury presentation [6]. Notably, these guidelines underwent modification during the COVID-19 era to alleviate NHS burdens, allowing pragmatic adaptation to the evolving healthcare landscape.

Change in guidelines during the COVID-19 pandemic 

The BOAST released an adjusted set of guidelines and recommendations for patients presenting with orthopaedic conditions during the pandemic [8]. The following quotes from BOAST highlight some of the important changes in the management of orthopaedic trauma [8]:

“Patients with complex fractures should have surgery planned to minimise the length of stay”. 

“Consider day-case treatment of simple peri-articular fractures and foot and ankle injuries”. 

“Where possible, use non-operative treatment and removable splints, recognising that some may require later reconstruction”. 

Since the release of these guidelines, there have been small-scale research conducted within the United Kingdom that aimed to analyse the impact of these changes and COVID-19 on foot and ankle fractures.

Current literature 

The existing body of literature predominantly addresses the repercussions of the COVID-19 pandemic, emphasising incident rates, patient demographics, and diverse facets of patient care. This study delves into the impact on surgically treated trauma cases admitted to the Liverpool Orthopaedic and Trauma Service during a 33-week period [9]. The analysis revealed a noteworthy decline in foot and ankle fractures, constituting 15.20% of all trauma cases pre-lockdown and diminishing to 8.81% during lockdown [9]. This indicates a significant reduction in the incidence of foot and ankle fractures during the pandemic [9], coupled with a notable shift towards conservative treatment approaches.

Concurrently, a study conducted at the University Hospitals of Leicester scrutinised patients with foot and ankle fractures, exposing a shift in demographics during lockdown [10]. Male patients' mean age decreased from 56 years pre-lockdown to 37 years during lockdown [10]. Surgical interventions witnessed an overall reduction, particularly for unstable fractures, aligning with a trend towards partial fixation in the theatre and direct discharge from the emergency department for stable fractures [10]. This paradigm shift in intervention strategies could potentially obviate the necessity for patient admission [10].

A national audit encompassing multiple UK centres investigated surgically treated foot and ankle patients across pre-lockdown, lockdown, and post-lockdown periods, elucidating a discernible reduction in overall surgical procedures during lockdown [11]. Elective surgeries, in particular, experienced a substantial decline, comprising 1.73% of activities during lockdown as opposed to 10.72% post-lockdown [11].

On an international scale, a comprehensive study in Sweden documented a statistically significant decrease in ankle fracture incidence during the pandemic [12]. This reduction, most prominent in the initial 30 days of the pandemic, disproportionately affected individuals aged >70 years and women [12], echoing findings from the local Liverpool study [9]. However, the absence of a full-scale lockdown in Sweden necessitates caution in generalising these results beyond the context of their unique societal response [12].

Aims 

The aim of this study is to analyse how the COVID-19 pandemic, lockdown, and subsequent change in guidelines have impacted patients admitted with foot and ankle fractures within Manchester University NHS Foundation Trust (MFT) hospitals. The parameters evaluated were fracture incidence, mean age, gender, and time from injury presentation until surgery. To our knowledge, there has been no prior study assessing how the COVID-19 pandemic has affected the mean time taken from injury presentation to surgery for foot and ankle fractures within the United Kingdom.

## Materials and methods

Data collection 

This is a retrospective observational study of data that was collected and screened using the Pathpoint™ eTrauma platform (Open Medical, London, UK). This system is utilised within MFT Hospital Sites: Manchester Royal Infirmary, Wythenshawe Hospital, and Trafford General Hospital. All admissions, referrals, and procedures regarding foot and ankle fractures from MFT hospitals are automatically added to the database. Data were cleaned and duplications were removed.

Inclusion and exclusion criteria 

The inclusion criteria were all adults (18+) admitted with foot and ankle fractures within five phases of time. The timeframe for each phase was taken from the official Institute for Government Analysis [13] (Table 1).

**Table 1:** Variation in phases by timeframe

Phase	Description	Dates	Days
Phase 0	Pre-pandemic	29/09/19 – 23/01/20	116
Phase 1	National lockdown 1	24/03/20 – 01/06/20	69
Phase 2	Post-lockdown: this timeframe was after the first national lockdown, where government restrictions were reduced.	15/06/20 – 14/10/20	121
Phase 3	National lockdown 2 (including a three-tier system)	15/10/20 – 2/12/20	48
Phase 4	National lockdown 3	06/01/21 – 12/04/21	96

Dates from January 23-March 24, 2020 were removed as the first case in the UK had been discovered but lockdown had not been initiated. This overlap meant COVID-19 could create a potential confounding variable within the pre-lockdown group, which was subsequently removed. Any period that did not fit into the national lockdown, three-tier system, or minimal restrictions post-lockdown were also removed.

Data analysis 

A mean was taken for the incidence rates and surgeries performed and presented per week to account for differences between the number of days within each phase. Both a range and a mean age were calculated for each phase and gender. A Poisson distribution was assumed for the incidence of foot and ankle fractures, and this enabled the calculation of confidence intervals (CI). Due to the local nature of the study and subsequent limitations of the sample size, 90% confidence intervals were chosen for the analysis of fracture incidence.

## Results

There were a total of 434 patients admitted with foot and ankle fractures in all five phases. Table 2 shows a breakdown of fracture incidence, mean age, and gender distribution within each phase.

**Table 2:** The distribution of patients admitted with foot and ankle fractures, their mean age, and gender by time period M: male; F: female

Admissions and patient demographics	Phase 0	Phase 1	Phase 2	Phase 3	Phase 4
	Pre-pandemic (116 days)	National lockdown 1 (69 days)	Post-lockdown (121 days)	National lockdown 2 (48 days)	National lockdown 3 (96 days)
Fracture admissions	N = 120	N = 49	N = 129	N = 43	N = 93
Fracture admissions per week (p/w)	7.24 p/w	4.97 p/w	7.46 p/w	6.27 p/w	6.78 p/w
Mean age and range	51.98 years (range: (18-93)	47.73 years (range: 19-97)	48.34 years (range: 18-87)	50.84 years (range: 18-102)	48.10 years (range: 18-97)
Gender distribution	M: 51	M: 24	M: 64	M: 21	M: 43
	F: 69	F: 25	F: 65	F: 22	F: 50
Mean age and range by gender	M: 45.76 years (range: 20-85)	M: 41.33 years (range: 21-77)	M: 43.72 years (range: 18-79)	M: 51.12 years (range: 18-78)	M: 43.09 years (range: 18-80)
	F: 56.58 years (range18-93)	F: 53.88 years (range: 19-97)	F: 52.89 years (range: 18-87)	F: 50.59 years (range: 21-102)	F: 52.22 years (range: 19-98)

**Figure 1:**
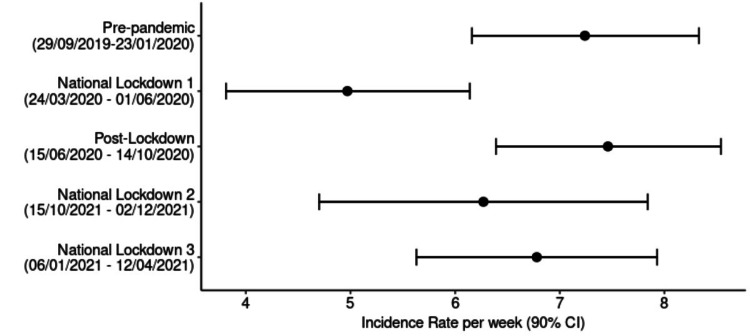
A forest plot of foot and ankle fracture admissions by time period.

From Table 2, a forest plot was created (Figure 1), and 90% confidence intervals were calculated in regard to the number of foot and ankle fracture admissions per week.

Table 2 provides a comprehensive overview of the mean fracture admissions per week during various phases, revealing notable trends in the context of national lockdowns. Throughout the observed periods, the mean fracture admissions per week were consistently lower during national lockdowns in comparison to both pre-pandemic and post-lockdown phases. Notably, national lockdown 1 exhibited a significant reduction, with only 4.97 fractures per week, while national lockdown 3 witnessed an increase to 6.78 per week. The pre-pandemic and post-lockdown phases displayed similar figures, maintaining averages of 7.24 per week and 7.46 per week, respectively.

In Figure 1, the 90% confidence intervals for each time period are illustrated, emphasising the statistical significance of certain observations. Specifically, there is no overlap between the confidence intervals for the pre-pandemic (6.16-8.13) or post-lockdown (6.39-8.54) groups with those of national lockdown 1 (3.81-6.14). This suggests a statistically significant reduction in foot and ankle fracture admissions during the initial national lockdown. Conversely, the second (4.7-7.84) and third national lockdowns (5.63-7.93) exhibit overlapping intervals with the pre-pandemic and post-lockdown phases, indicating a lack of statistical significance.

Gender distribution reveals a consistent predominance of female patients across all phases, with the greatest difference observed during the pre-pandemic phase (69 females and 51 males admitted). Interestingly, the mean age of patients in the pre-pandemic cohort was notably higher at 51.98 years, surpassing the averages of all other groups. Additionally, females, on average, were older than their male counterparts in all phases except during national lockdown 2.

In Table 3, the weekly incidence of surgeries closely mirrors the fracture trends, peaking in the post-lockdown phase at 6.31 surgeries per week and reaching a nadir during national lockdown 1 at 4.77 per week.

**Table 3:** Surgical procedures conducted on patients with foot and ankle fractures within each phase of time *A delayed surgical procedure is defined as a procedure that was cancelled and re-booked for a forward date.

Surgical procedure details	Phase 0	Phase 1	Phase 2	Phase 3	Phase 4
	Pre-pandemic (116 days)	National lockdown 1 (69 days)	Post-lockdown (121 days)	National lockdown 2 (48 days)	National lockdown 3 (96 days)
Number of surgical procedures	N = 98	N = 47	N= 109	N = 39	N = 84
Surgical procedures per week (p/w)	5.91 p/w	4.77 p/w	6.31 p/w	5.69 p/w	6.12 p/w
Number of surgical procedures delayed*	48 delays	15 delays	40 delays	14 delays	25 delays
Mean wait time from injury presentation to surgery (days)	8.74 days	7.11 days	7.56 days	7.23 days	5.79 days

Remarkably, the third national lockdown witnessed a resurgence to 6.12 per week, surpassing the pre-pandemic rate of 5.91 per week. The mean interval from injury presentation to surgery exhibited a disparate trajectory. The pre-pandemic cohort experienced the longest wait time at 8.74 days, concurrent with 48 delayed surgeries. Conversely, national lockdown 3 demonstrated the shortest mean wait time of 5.79 days, coupled with only 25 delays.

## Discussion

Incidence of foot and ankle fractures 

Foot and ankle fracture admissions were highest among the pre-pandemic and post-lockdown groups and lower during national lockdowns. This suggests that lockdown, adjustments to BOAST guidelines, and increased government restrictions correlate with a decrease in foot and ankle fracture admissions. However, each lockdown appeared to be less effective, as the number of fractures per week increased with each successive lockdown. This result is consistent with studies conducted both within the United Kingdom and Sweden, which showed an initial large decrease in foot and ankle fracture incidence and then a subsequent gradual increase [9-10, 12]. This effect may be explained by research showing that people find it more difficult to comply with lockdown restrictions the longer they are enforced [14].

Patient demographics 

The mean age of patients presenting with foot and ankle fractures was highest within the pre-pandemic cohort. The reduction in mean age post-pandemic is unsurprising, given that UK government guidance states that older patients are at higher risk and should be advised to stay at home where possible [15]. The government's advice and suggested restrictions for the elderly population may contribute to a less active lifestyle and a subsequent reduction in fracture incidence.

Furthermore, the ratio of female to male patients was higher pre-pandemic than within any other phase post-COVID-19. The decrease in the ratio of female to male patients post-COVID-19 may be due to a multitude of reasons. The study within Sweden, which found similar results, theorised that the reduction in female foot and ankle fractures may be due to women adhering to government guidelines more closely [11]. This explanation is supported by statistics, as an article from the British Broadcasting Corporation (BBC) quoted data from the National Police Chief’s Council showing that eight out of 10 people breaking lockdown regulations were males [15].

Surgical procedures and mean wait time 

The frequency of surgical procedures mirrored the trends observed in foot and ankle fracture incidence across different pandemic phases. As anticipated, the initial national lockdown yielded the fewest surgeries due to the cancellation of elective procedures and a decline in foot and ankle fracture admissions. In contrast to fracture rates, both the post-lockdown and the third national lockdown exhibited higher weekly rates of surgeries compared to the pre-pandemic period. This surge in surgical procedures after lockdowns may be attributed to a backlog resulting from the initial restrictions. Acknowledging this, the BOAST recognised the potential for increased demand, with some cases requiring delayed reconstruction [8]. Interestingly, a study from the Liverpool Orthopaedic and Trauma Service demonstrated a rebound in all surgically treated lower limb trauma except for foot and ankle fractures [9].

The mean wait time from injury presentation to surgery was shortest during the third national lockdown and longest in the pre-pandemic phase. This reduction in waiting time during the third lockdown may be linked to modified BOAST guidelines advocating for minimal hospital stays and potential increased hospital efficiency as staff adapted to the altered protocols. However, comprehensive research from diverse centres is imperative to draw definitive conclusions regarding the observed trends.

Limitations 

There are some limitations to this study. The analysis of foot and ankle fractures began in September 2019, as this is when the MFT hospitals switched to the eTrauma system. Ideally, multiple previous years would be used as a control. This would enable the study to account for any potential seasonal impact or identify outlier time periods. Furthermore, this is a single-trust study, and therefore, this may limit the generalizability of the results. There is potential for this study to be extended to further regions throughout the UK.

## Conclusions

In conclusion, the study demonstrates that the COVID-19 pandemic and lockdown have had a significant impact on the incidence of foot and ankle fracture admissions, patient demographics, and surgical procedures performed. The incidence of both fractures and surgical procedures decreased significantly during the first national lockdown, with a gradual increase in the second and third lockdowns. The number of surgical procedures performed per week increased during the third national lockdown due to the backlog of procedures. Finally, the mean time from injury presentation was reduced post-COVID-19 and was lowest during the third national lockdown.

## References

[1] (2023). Rolling updates on coronavirus disease (COVID-19). https://www.who.int/emergencies/diseases/novel-coronavirus-2019/events-as-they-happen.

[2] (2023). Prime Minister's statement on coronavirus (COVID- 19): 23 March. https://www.gov.uk/government/speeches/pm-address-to-the-nation-on-coronavirus-23-march-2020..

[3] Myles PS, Maswime S (2020). Mitigating the risks of surgery during the COVID-19 pandemic. Lancet.

[4] Propper C, Stoye G, Zaranko B (2020). The wider impacts of the coronavirus pandemic on the NHS. Fisc Stud.

[5] (2023). COVID-19 coronavirus pandemic. https://www.worldometers.info/coronavirus/.

[6] (2023). BOAST - the management of ankle fractures. https://www.boa.ac.uk/resource/boast-12-pdf.html.

[7] (2023). Fractures (non-complex): assessment and management. https://www.nice.org.uk/guidance/ng38/evidence/full-guideline-2358460765.

[8] (2023). Management of patients with urgent orthopaedic conditions and trauma during the coronavirus pandemic. https://www.boa.ac.uk/static/782e0b20-f9ce-4fc9-819f943740161405/201ebd61-5828-4c81-b45a8b80ac47fd50/COVID-19-BOASTs-Combined-v3FINAL.pdf.

[9] Stringer H, Molloy A, Craven J, Moorehead J, Santini A, Mason L (2021). The impact of COVID-19 on foot and ankle surgery in a major trauma centre. Foot (Edinb).

[10] Shah R, Ahad A, Faizi M, Mangwani J (2021). Foot and ankle trauma management during the COVID-19 pandemic: experiences from a major trauma unit. J Clin Orthop Trauma.

[11] Mason LW, Malhotra K, Houchen-Wollof L, Mangwani J (2022). The UK foot and ankle COVID-19 national (FAlCoN) audit - regional variations in COVID-19 infection and national foot and ankle surgical activity. Foot Ankle Surg.

[12] Rydberg EM, Möller M, Ekelund J, Wolf O, Wennergren D (2021). Does the Covid-19 pandemic affect ankle fracture incidence? Moderate decrease in Sweden. Acta Orthop.

[13] (2021). Timeline of UK coronavirus lockdowns, March 2020 to March 2021. March.

[14] Simon Halliday, Jed Meers, and Joe Tomlinson (2023). Simon Halliday, Jed Meers, and Joe Tomlinson: public attitudes on compliance with Covid-19 lockdown restrictions. https://ukconstitutionallaw.org/2020/05/08/simon-halliday-jed-meers-and-joe-tomlinson-public-attitudes-on-compliance-with-covid-19-lockdown-restrictions/.

[15] (2023). Coronavirus: more than 9,000 fines for lockdown breaches. https://www.bbc.com/news/uk-52489943.

